# Relationship between admission criteria and academic performance: A correlational study in nursing students

**DOI:** 10.12669/pjms.35.3.217

**Published:** 2019

**Authors:** Imran Inayat Yousafzai, Brekhna Jamil

**Affiliations:** 1 *Mr. Imran Inayat Yousafzai, BSN, MSPH, MHPE., Khyber Medical University, Peshawar, Pakistan*; 2 *Dr. Brekhna Jamil, BDS, MPH, MHPE. Assistant Professor, Institute of Health Professions Education & Research, Khyber Medical University, Peshawar, Pakistan*

**Keywords:** Admission criteria, academic performance, Post RN, Nursing

## Abstract

**Background & Objective:**

An admission criterion is one of the key indicators of academic success. The purpose of admission process is to select best candidates for the particular program who will complete the program successfully. Thus rigorous admission criteria can predict academic performance, decrease failure rate and successful completion of degree. The objective of this study was to determine the relationship between various variables in the existing admission criteria and academic performance.

**Methods:**

A cross sectional study design was chosen. Data (2009-2017) was gathered from the records of Institute of Nursing Science, Khyber Medical University. Variables in the admission criteria included age, gender, previous academic performance, entry test score and experience. The dependent variable was academic performance measured in CGPA. The data was analyzed using correlation and regression analysis through SPSS and STATA.

**Results:**

The results reported a significant relationship between admission criteria and the academic performance of nursing students. Various variables in the admission criteria i.e. SSC marks (r=0.32, p=>0.001), previous academic score at diploma level (r=0.48, p=>0.001) and entry test scores (r=0.26, p=>0.001) have significant relationship between academic performance. However, previous academic scores at diploma level were better predictors of the academic performance.

**Conclusion:**

The study concludes to use integrated admission criteria for the selection of students and bring changes in the traditional admission process.

## INTRODUCTION

Admission criteria is one of the key indicators to predict academic performance.^1^ Educationists are interested in identifying key factors which can predict academic performance. Studies suggest that rigorous validated admission criteria can predict the decrease failure rate and lead to successful completion of program.^2^

Literature reviews suggests that admission criteria constitutes of cognitive and non-cognitive factors.^3^ Cognitive variables include aptitude tests, previous academic marks, admission tests, while non-cognitive factors includes gender, previous experience, age, race, personality and ethnicity. Various cognitive and non-cognitive factors are set in the admission criteria to identify and select students who can successfully complete their programs.^4^,^5^

Available evidence suggests that a better formulated admission criteria including mix of academic and non-academic factors can predict better academic performance.^6^ Admission criteria to diplomas and baccalaureate degree in nursing in Pakistan mainly consist of previous academic performance and entry tests score, age, gender and interviews. Admission criteria have not been researched to inform whether it is a strong predictor of academic performance. The purpose of this study was to assess the relationship between admission criteria and academic performance in the Post RN BSN nursing students at Khyber Medical University, Peshawar.

## METHODS

A correlational study was conducted. A specifically designed Checklist was used to record the data from the available records on admission criteria and academic performance. The checklist includes the information about Marks in SSC, Previous academic Marks in General nursing and midwifery/speciality, Entry Test Marks, Academic performance in GPA. It was validated from the expert in the field of medical education and nursing education. The checklist was also field tested for errors before data collection. The records of all 197 Post RN nursing students enrolled and passed out between (2008-2017) in Khyber Medical University, Peshawar was reviewed using the checklist. Those who failed to complete the course due to any reason were not included in the study. Data was collected after ASRB and ethical approval from ethical review board of Khyber Medical University. Data regarding enrolment characteristics was collected from the students’ personal files and admission records while data about academic performance in Post-RN Program were obtained from examination section of Khyber Medical University, Peshawar. The study variables included previous academic marks measured in percentage obtained in Matric (higher secondary school certificate), Nursing diploma, entry test marks, gender and age of the student at the time of admission. Outcome variable was academic performance that was measured in CGPA. Descriptive analysis was done on all 197 participants. Regression analysis, t-test and Pearson’s correlation analysis were also done. Data analysis was carried out using SPSS version 22.0 and STATA.

## RESULTS

Results showed that majority of the participants 86.3% (n=170) were females. The mean ages of the participants were 34.33 +- SD5.66. This study shows a significant (p<0.001) difference (0.29) in the mean academic performance of males and females. This difference is statistically significant (p>0.001) after using t test. Apart from this, a moderate relationship (r=0.32, p<0.001) was observed between SSC marks and academic performance. A moderate relationship (r=0.48, p<0.001) was also observed between the previous academic score and the academic performance. The results show a significant weak relationship between academic performance and entry test score (r=0.26, p=<0.001) and also between age at the time of admission and the academic performance (r= -0.15, p=0.030), besides weak non-significant negative relationship between years of experience and the academic performance of the nursing students (r= -0.09, p=>0.18). Table-I shows a detailed statistical analysis of correlation.

**Table-I:** Relationship of academic performance with variables of admission criteria using Pearson’s correlation analysis (N=197).

	Age at the time of admission	SSC Marks	Previous academic score in diploma nursing	Entry Test score	Experience
Academic	-0.1539*	0.3287*	0.4825*	0.2698*	-0.0953
Performance	0.0308	0.0000	0.0000	0.0001	0.1827

Dependent variable CGPA, coded

* p <0.05

As shown in Table-II, regression analysis was done between the admission criteria and the academic performance. The predictor variables include gender, age at the time of admission, SSC marks, previous academic marks in nursing diploma, entry test score and years of experience whereas the dependent variable is academic performance measured in CGPA. The results of regression model shows that the previous academic score at diploma nursing level (β=0.0327, p<0.001), SSC marks (β=0.0127, p<0.001) and years of experience (β=0.0237, p<0.011), entry test score (β=0.005, p<0.002) were better predictor of academic performance in nursing students. However, age at the time of admission recorded in years (β= -0.018, p<0.019) and the gender being female (β= -0.164, p<0.001) were statistically significant negative predictors with the academic performance of the students.

**Table-II:** Regression analysis for variables predicting academic performance. (N=197)

Predictor variables	Academic performance		

	coefficient (β)	(95 CI)	P value
Gender			
Male*	-0.1642	(-0.3014, -0.0270)	0.019
Female			
Age at admission	-0.0181	(-0.3185, -0.0043)	0.010
SSC Marks	0.0127	(0.0063, 0.019)	0.000
Marks at diploma Nursing	0.0327	(0.0242, 0.0412)	0.000
Entry Test score	0.0052	(0.0019, 0.0085)	0.002
Experience	0.0237	(0.0054, 0.04197)	0.011

## DISCUSSION

The results of this study has shown a significant weak relationship between SSC marks and the academic performance (r=0.32, p=<0.001) and also between the previous academic score at diploma nursing and the academic performance (r=0.48, p=<0.001). These results are consistent with previous research studies which support the notion that previous academic performance could predict subsequent academic performance of a student.^2^,^3^,^7^-^9^

A significant but weak relationship (r=0.26, p=<0.001) was observed between academic performance and entry test score. However, previous academic score at diploma nursing level (β=0.0327, p<0.001), SSC marks (β=0.0127, p<0.001), years of experience (β=0.0237, p<0.011) and entry test score (β=0.005, p>0.002) were better predictor of a good academic performance of the post RN nursing students academic performance. The findings of this research study confirmed the relationship between admission entrance test and the academic performance as in the literature.^10^,^11^ However, there are other research studies that have shown either a poor or no predictive relationship between entrance tests and academic performance in health sciences students. In contrast to these findings, various researches have also shown no relationship between the aptitude test and initial academic performance.^12^ Entrance test alone is itself a weak predictor of the academic performance, however it can provide better screening if it is used jointly with other measures.^10^ Moreover, it is also suggested that an integrated approach may be used in the selection process with further researches.^13^,^14^

This study also shows a significant weak negative relationship (r= -0.15, p=0.030) between age and the academic performance, whereas age is a negative predictor (β= -0.018, p<0.019) of the academic performance. The result of this study is partly in contrast and partly in lined with other studies that old age is negatively co-related to academic performance.^15^,^16^ In addition to this, as age increases the academic performance may actually decline. Other research studies have also shown similar findings that younger students perform better than those who are old.^17^

This study shows a significant (p<0.001) difference (0.29) in the mean academic performance of males and females. This difference is statistically significant. A research study by Ali et al. reported that female gender is negatively co-related with academic performance. However, the co-relation is insignificant.^7^ In contrast to this, another research study has shown that the academic performance of females nursing students is higher than the male students.^18^ The regression analysis of this research study has shown that gender being female (β= -0.164, p<0.001) is negative predictors with the academic performance. However, research findings has revealed gender being female was a significant predictor of academic performance.^18^ In addition to this, there are various research studies that have shown no significant difference in academic achievement between male an-d female students.^19^,^20^

## CONCLUSION

The results of this study have shown significant relationship of various variables in the existing admission criteria with the academic performance that can be used to select nursing students who can be academically successful in future. Previous academic score at diploma nursing level, SSC marks, years of experience and entry test score were better predictor of the academic performance in nursing students. Moreover, the study concludes a significant relationship of academic performance with SSC marks, previous academic score at diploma nursing and entry test score. This study recommends using an integrated approach of selecting students for the post RN nursing program. However, different variables in the integration should be given weightage in accordance with the strength of relationship between the variables and the academic performances. The study may be replicated to other provinces and disciplines of health professions education programs so that a debate can be generated for rigorous admission criteria and changes in the current admission criteria.

### Authors’ Contribution

**IIY:** Conceived, Designed, Data collection and did statistical analysis & manuscript writing.

**BJ:** Did review, Editing of manuscript and final approval of manuscript.
